# Comparison of microRNA profiles between benign and malignant salivary gland tumors in tissue, blood and saliva samples: a prospective, case-control study^☆^

**DOI:** 10.1016/j.bjorl.2016.03.013

**Published:** 2016-04-27

**Authors:** Ovgu Cinpolat, Zeynep Nil Unal, Onur Ismi, Aysegul Gorur, Murat Unal

**Affiliations:** a Gaziantep State Hospital, Gaziantep, Turkey; b University of Mersin, Faculty of Pharmacy, Department of Biochemistry, Mersin, Turkey; c University of Mersin, Faculty of Medicine, Department of Otorhinolaryngology, Mersin, Turkey; d University of Mersin, Faculty of Medicine, Department of Medical Biochemistry, Mersin, Turkey

**Keywords:** Salivary gland tumor, MicroRNA, miR-21, miR-30e, Pathogenesis, Tumor de glândula salivar, MicroRNA, miR-21, miR-30e, Patogenia

## Abstract

**Introduction:**

Salivary gland tumors (SGTs) are rare head and neck malignancies consisting of a spectrum of tumors with different biological behaviors.

**Objective:**

In this study we aimed to find out differential expression of microRNA profiles between benign and malignant SGTs.

**Methods:**

We investigated the possible role of 95 microRNAs in the 20 patients with salivary gland tumors with comparison of 17 patients without malignancy or salivary gland diseases. Sixteen of the tumors were benign (seven pleomorphic adenomas, nine Warthin tumors), four of them were malignant (two squamous cell carcinomas, one high grade mucoepidermoid carcinoma, one adenocarcinoma). Serum and saliva samples were collected from both patients and control group. Tissue samples of tumor masses were also collected from patient group.

**Results:**

Among studied microRNAs miR-21, miR-23a, miR-27a, miR-223, miR-125b, miR-126, miR-146a, miR-30e were down regulated in the benign group compared to control group in the serum samples (*p*-values are 0.04, 0.00005, 0.00005, 0.0022, 0.031, 0.00008, 0.044, and 0.0007, respectively). When tissue samples were studied miR-21, miR-31, miR-199a-5p, miR-146b, miR-345 were up-regulated in the malignant group compared to benign group (*p* values are 0.006, 0.02, 0.013, 0.013, 0.041, respectively). miR-30e showed statistically significant up-regulation in malignant tumor group's plasma samples compared to benign group (*p* = 0.034). There was no statistically significant difference in saliva samples between groups.

**Conclusion:**

Our results showed that different microRNAs may play role in salivary tumor pathogenesis according to biological behavior. Although there was no difference in saliva samples between groups, according to tissue and serum samples miR-21 and 30e may have an important role; since they were down-regulated in benign tumors whereas up-regulated in malignant ones.

## Resumo

**Introdução:**

Os tumores da glândula salivar (TGS) são lesões malignas raras de cabeça e pescoço que consistem em um espectro de tumores com diferentes comportamentos biológicos.

**Objetivo:**

Neste estudo, tivemos como objetivo identificar a expressão diferencial de perfis de microRNA entre TGS benignos e malignos.

**Método:**

Investigamos a possível participação de 95 microRNA em 20 pacientes com tumores de glândulas salivares comparado a 17 pacientes sem doença maligna ou doenças das glândulas salivares; 16 dos tumores eram benignos (sete adenomas pleomórficos, nove tumores de Warthin), quatro deles eram malignos (dois carcinomas espinocelulares, carcinoma mucoepidermoide de alto grau, um adenocarcinoma). As amostras de soro e saliva foram coletadas de pacientes e do grupo controle. Amostras de tecido dos tumores também foram colhidas do grupo de pacientes com tumores.

**Resultados:**

Entre os microRNA estudados, miR-21, miR-23a, miR-27a, miR-223, miR-125b, miR-126, miR-146a, miR-30e foram infrarregulados no grupo benigno em comparação com o grupo controle nas amostras do soro (os valores de *p* são 0,04, 0,00005, 0,00005, 0,0022, 0,031, 0,00008, 0,044 e 0,0007, respectivamente). Quando as amostras de tecido foram estudadas, miR-21, o miR-31, o miR-199-5p, miR-146b, o miR-345 foram suprarregulados no grupo maligno em relação ao grupo benigno (valores de *p* são 0,006, 0,02, 0,013, 0,013, 0,041, respectivamente). O miR-30e apresentou suprarregulação estatisticamente significativa em amostras de plasma do grupo de tumor maligno em relação ao grupo benigno (*p* = 0,034). Não houve diferença estatisticamente significativa em amostras de saliva entre os grupos.

**Conclusão:**

Nossos resultados mostraram que diferentes microRNA podem desempenhar um papel na patogenia do tumor salivar de acordo com o comportamento biológico. Embora não tenha havido diferença em amostras de saliva entre os grupos, de acordo com as amostras de tecido e de soro, miR-21 e 30e podem ter um papel importante, já que foram infrarregulados nos tumores benignos enquanto suprarregulados nos tumores malignos.

## Introduction

Salivary gland tumors (SGT) comprise only 3–5% of all head and neck malignancies; they have at least 24 different types according to World Health Organization 2005 classification.^1^ Among these pleomorphic adenoma is the most common benign tumor whereas mucoepidermoid carcinoma is the most common malignant one.^2^ The exact pathogenesis and way to malignant transformation for salivary gland tumors are not well known. Although cigarette smoking and alcohol consumption are important risk factors for head and Squamous Cell Carcinomas (SCC), they are not admissible for salivary gland tumors, and occupational exposures are unlikely to take part in the pathogenesis.^3^

MicroRNAs (miRNAs) are a group of endogenous 21–25 nucleotide noncoding RNAs which target gene coding in the posttranscriptional level.^4^, ^5^ They are involved in various important biological processes such as development, differentiation, proliferation and apoptosis.^5^ They can behave like oncogenes or tumor suppressor genes; their up regulation or down regulation may take part in carcinogenesis.^6^ Among head and neck cancers SCC and among salivary gland malignancies Adenoid Cystic Carcinomas (ACCs) are the most common cancers studied for possible role of miRNAs in cancer pathogenesis.^4^, ^5^, ^6^, ^7^ In this study we investigated the possible role of miRNAs in salivary gland tumor pathogenesis. To the best of our knowledge; this is the first study with comparing serum, saliva and tissue levels of miRNAs among salivary gland neoplasms.

## Methods

Local ethical committee approval was acquired for our study. 20 patients with salivary gland tumors and 17 sex and age matched healthy controls without any salivary gland or systemic disorders were included. Mean age for salivary gland tumor was 53.1 and mean age for control group was 46.4. There were 10 male (50%) and 10 female (50%) patients in tumor group and 9 male (52.9%) and 8 female (47.1%) patients in the control group. There was no statistically significant difference regarding age (*p* = 0.251) and sex (*p* = 0.858) between tumor and control groups. From the tumor group there were 16 benign and 4 malignant tumors. Malignant ones were two SCCs, one high grade mucoepidermoid carcinoma, one adenocarcinoma. Only the primary squamous cell carcinomas were taken to the study according to Gaughan's inclusion criteria,^8^, ^9^ metastatic squamous cell carcinomas were excluded. Benign ones were nine Warthin tumors and seven pleomorphic adenomas. List of the tumors were summarized in Table 1. In the tumor group one (25%) of four in the malignant group and five (31.25%) of 16 in the benign group were female. Regarding sex and age differences between malignant and benign tumor groups, there was no statistically different for both sex (*p* = 0.807) and age (*p* = 0.9355).

**Table 1:** Histopathologic types of tumors in the patient group.

Tumor type	Percentage
Warthin's tumor	9/20 (45%)
Pleomorphic adenoma	7/20 (35%)
Squamous cell carcinoma	2/20 (10%)
High grade mucoepidermoid carcinoma	1/20 (5%)
Adenocarcinoma	1/20 (5%)

### MicroRNA analysis

#### Serum collection

Blood samples of 5 mL were collected to 7.5% EDTA containing tubes from patient group preoperatively and control group. After centrifugation serums were collected and stored at −80 °C.

#### Saliva collection

After catheterization of the involved salivary gland ductus, by using lemon as sialogogue 200 μL saliva of the involved gland was collected. The saliva was mixed with 200 μL RNAlater (QIAGEN Inc., Valencia, CA) solution. After two hours in room temperature, they were stored at −80 °C in deep freezer.^10^ 200 μL saliva was also collected from the control group.

#### Tissue collection

Tissue samples were taken only from the salivary gland tumor group. During surgery for salivary gland tumor; a 3–4 mm^3^ tumoral tissue was used for analysis. Tissue samples were put into 1 mL RNAlater (QIAGEN Inc., Valencia, CA) solution. After two hours in room temperature, they were stored at −80 °C in deep freezer. Tissues were diced with a surgical blade and homogenized with pestle and mortar. MicroRNA isolation from the homogenized tissue was done by MicroRNA isolation kit (Roche Diagnostics, GmbH, Mannheim, Germany) according to manufacturer's instructions.

#### MicroRNA expression profiling

After MicroRNAs were isolated from serum, saliva and tissue by MicroRNA isolation kit (Roche Diagnostics, GmbH, Mannheim, Germany) according to manufacturer's instructions; RNA samples converted to cDNA by using miScript II RT Kit (Qiagen). cDNA samples are preamplified by using miScript Microfluidics PreAMP Kit (Qiagen). qRT-PCR analysis performed by using miScript miRNA Assays (Qiagen) with Dynamic Array 96.96 (Fluidigm) on BioMark System (Fluidigm). Basically 95 types of microRNAs were studied.

#### Data analysis

qRT-PCR results analyzed by using 2^−ΔΔCt^ method.^11^, ^12^ Briefly, up or down regulation of microRNAs were compared between groups. Student's *t*-test was used for analyzing age differences and Chi-square test was used for analyzing sex differences between groups. Expression data were controlled for normal distribution with the Shapiro–Wilk test. According to the results; all data were not normally distributed. Mann–Whitney *U*-test was used to detect expression differences of miRNAs in serum, tissue and saliva samples. *p*-Value of <0.05 was considered as statistically significant.

## Results

**Figure 1:**
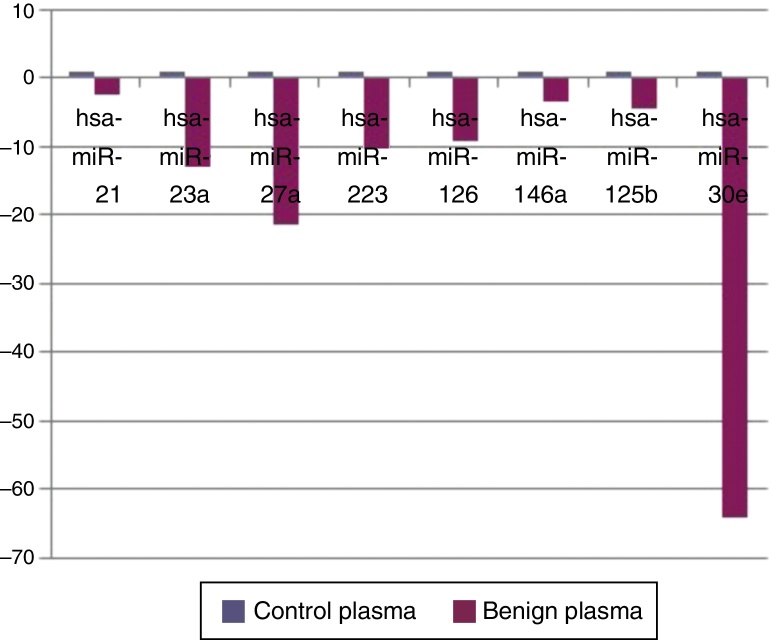
Comparison of plasma microRNA expression profiles between benign tumor group and control group.

When benign tumors were compared with control group; 8 miRNAs (miR-21, miR-23a, miR-27a, miR-223, miR-125b, miR-126, miR-146a, and miR-30e) as shown in Fig. 1, had statistically significant down-regulation in benign tumor group in serum samples. Table 2 summarizes the fold regulation and *p*-values of these miRNAs. There was no statistically significant up-regulation of miRNAs in serum samples.

**Table 2:** MicroRNAs showing statistically significant fold changes by comparing the plasma samples of benign tumor and control groups with *p* values were presented.

MicroRNA type	Fold regulation	*p*-Values
Mir-21	−2.5197	0.04
Mir-23a	−13.05	0.00005
Mir-27a	−21.4	0.00005
Mir-125b	−4.644	0.031
Mir-126	−9.2	0.00008
Mir-30e	−64.38	0.0007
Mir-146a	−3.5	0.044
Mir-223	−10.34	0.0022

**Figure 2:**
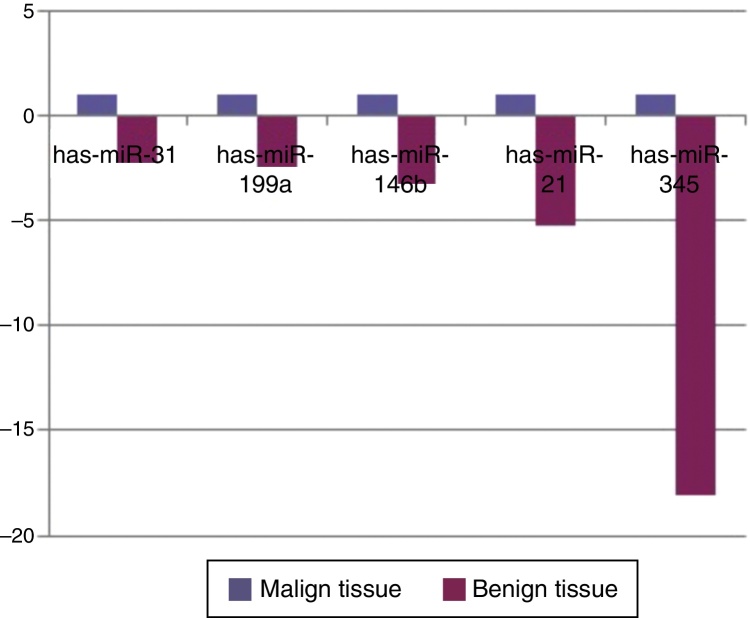
Comparison of tissue microRNA expression profiles between benign and malignant tumor groups.

**Figure 3:**
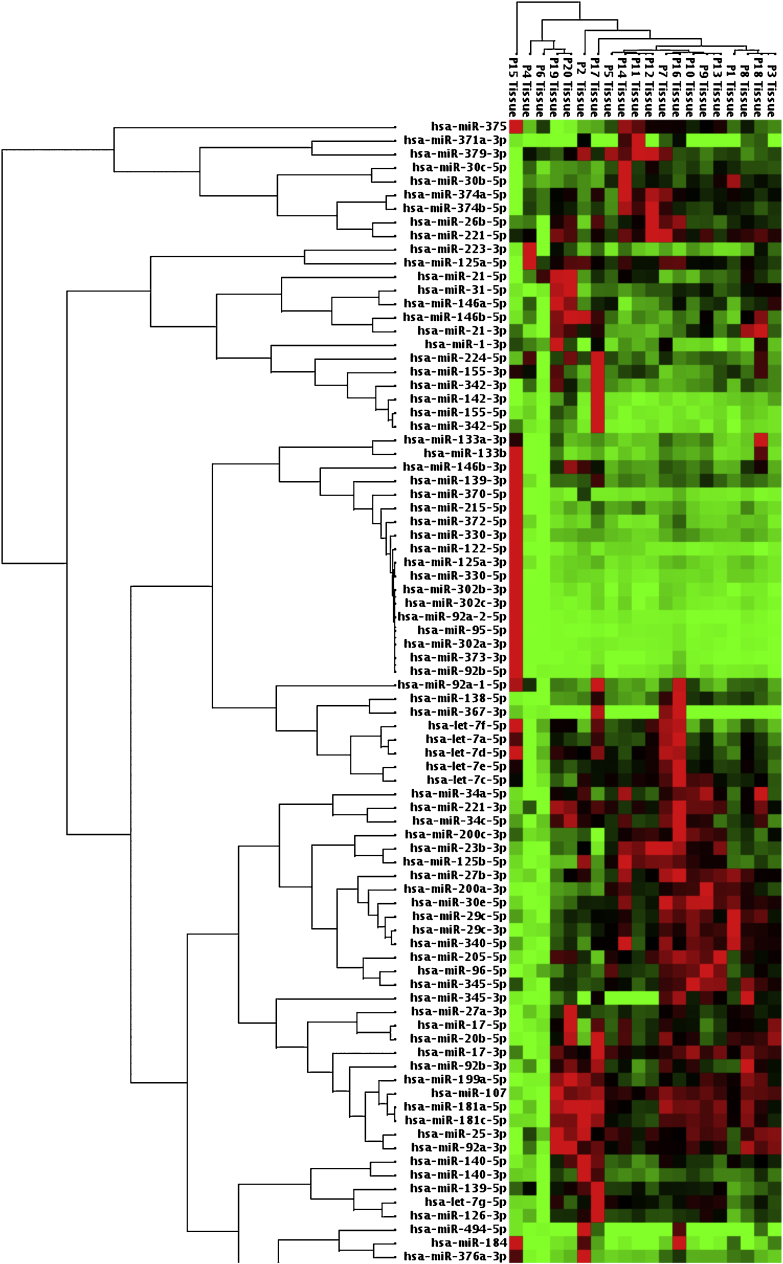
Heat map diagram of patients’ microRNA expression profiles in tissue samples was presented. P (patient) 8, 9, 18, 20 were malignant salivary gland tumor patients, others were benign ones.

**Figure 4:**
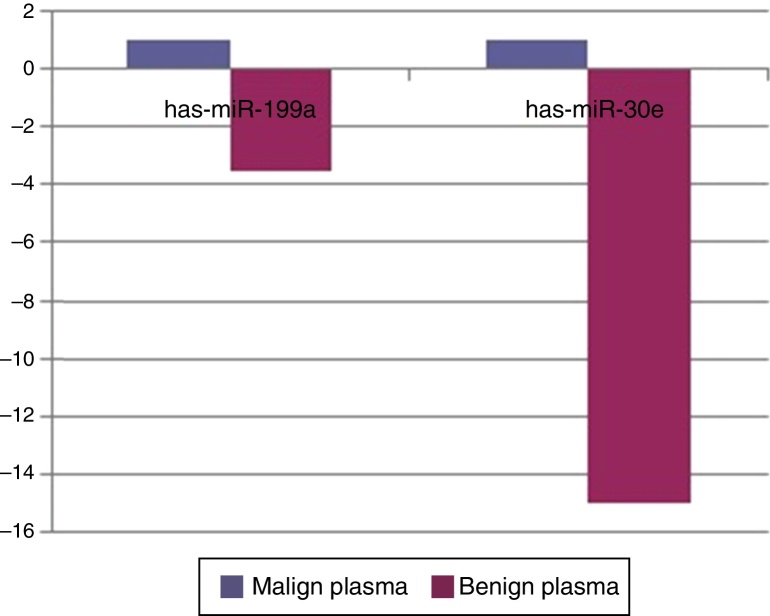
Comparison of plasma samples of benign and malignant tumor group for microRNA expression profiles.

When malignant tumor group patients were compared with benign tumor group in tissue samples; 5 miRNAs (miR-21, 31, 199a, 146b, 345) showed statistically significant up-regulation in malignant tumor group (Fig. 2). Table 3 summarizes the fold regulation values in malignant tumor group with *p*-values. Heat map diagram of clustering of miRNAs in tissue samples were shown in Fig. 3. Among these miRNAs miR-199a also showed statistically significant (*p* = 0.042) up-regulation in serum samples of malignant group compared to benign group. miR-30e showed 15.06 fold up-regulation (*p* = 0.034) in malignant tumor group's plasma samples compared to benign group (Fig. 4).

**Table 3:** MicroRNA expression of malignant tumor group compared to benign tumor group in tissue samples showing statistically significant fold changes were shown.

MicroRNA type	Fold regulation	*p*-Values
Mir-21	+5.23	0.0006
Mir-31	+2.24	0.02
Mir-199a	+2.36	0.013
Mir-146b	+3.21	0.013
Mir-345	+18.063	0.041

When malignant tumor group was compared with control group miR-23a showed 10.5 fold down regulation (*p* = 0.02) in serum samples of malignant tumors (data not shown).

There was no statistically significant difference in saliva samples between benign, malignant and control groups.

## Discussion

Since they were first found in the nematode *Caenorhabditis elegans* in 1993,^13^ miRNAs have been investigated for their probable roles in normal physiological and diseased states with several studies. They are a class of endogenous small RNAs which target gene expression in the posttranscriptional level that negatively regulate messenger RNA translation.^4^, ^14^ After transcripted via RNA polymerase II or III, they have multiple important biological behaviors such as proliferation, differentiation, apoptosis and motility.^4^, ^14^ These important features make them crucial in physiological development of tissues and anomalies in their transcription may lead to aberrant differentiation and carcinogenesis.^6^

For normal embryological development of salivary glands, roles of miRNAs have been investigated by authors. In a murine model Jevnaker and Osmundsen^15^ found that there were tissue specific miRNAs in the submandibular gland for normal development such as has-miR-28, 150, 222, 299, 322, 329, 341, 375 and 429. miRNAs miR-23a, 27a, 223, 125b, 126, 30e which were shown to be down regulated in benign tumor group in our study were also expressed in submandibular gland of mice embryo. Also some members of miRNA 17–92 cluster such as miR-19b, miR-20a, miR-92 were highly expressed during mice submandibular gland development.^14^ miR-21 which was one of the most important miRNAs studied in our report, is also a crucial miRNA for embryological development of submandibular gland and its branching morphogenesis. It was shown to be expressed in mesenchymal tissue of mice submandibular gland, this expression was up-regulated by epidermal growth factor which has critical role in branching of submandibular gland ductus.^16^

Near normal physiological development roles of miRNAs in carcinogenesis are also investigated. Since the first signs of their probable role in carcinogenesis were found by Calin et al. ^17^ by down-regulation of miR-15a, miR-16-1 in chronic lymphoid leukemia in 2002, several studies concerning relationship between miRNAs and carcinogenesis have been published. Among head and neck cancers squamous carcinoma is widely studied for probable role of miRNAs. Although there is a wide difference between studies, miR-21 is the only constant miRNA showing up regulation in head and neck squamous cell cancers.^6^ It has anti-apoptotic, cell proliferative effects with promoting cell invasion and metastases, so up regulation of this miRNA also predicts poor prognosis in head and neck cancers.^18^ In our study there was a fivefold (*p* = 0.0006) up-regulation of miR-21 in malignant salivary gland tumors’ tissue samples compared to benign tumors. There was also a 2.51 fold down-regulation (*p* = 0.047) in plasma samples of benign group compared to control group. This miRNA may have a critical role in both benign and salivary gland tumor pathogenesis.

In our study miR-125b was shown to be down regulated in serum samples of benign salivary gland tumors. miR-125b and miR-100 are two important miRNAs mapping the chromosome 11. Alterations in this chromosome may lead to both oral cancers and mucoepidermoid carcinoma of salivary gland.^19^, ^20^ In the study of Hui et al.^18^ expression of miR-125b was also down regulated in head and neck squamous cell carcinoma like our study. Down regulation of this miRNA may cause increased cell proliferation and carcinogenesis.^19^

MiR-23a and miR-27a belong to miR 23a∼27a∼24-2 cluster located on chromosome 9q22. They have multiple functions in both healthy and disease states such as cell cycle, proliferation, differentiation and cardiac hypertrophy.^21^ Literature investigating the role of these miRNAs in carcinogenesis is conflicting. miR-23a and miR-27a was shown to be down regulated in oral SCC,^22^ whereas recently Peng et al. showed that miR-23a promotes chemoresistance to cisplatin chemotherapy in tongue SCC.^23^ miR-23a and 27-a are also down regulated in acute promyelocytic leukemia whereas they are up regulated in acute myeloid leukemia, acute lymphoblastic leukemia, gastric cancer and hepatic cancer.^21^ In the light of recent literature it is obvious that these miRNAs may behave different in pathogenesis of different cancers, they may act as tumor suppressor gene in one cancer while oncogene in another. In our study miR-23a and 27a were shown to be down regulated in serum samples of benign salivary gland tumors. miR-23a was also down regulated in malignant tumor group compared to control group in serum samples. miR-23a may behave like a tumor suppressor gene in both benign and malignant salivary gland tumors.

When tissue and plasma samples were compared among benign and malignant salivary gland tumors in our study; there was a 2.3656 fold up-regulation (*p* = 0.01357) of miR-199a-5p in tissue samples and 3.5 fold up regulation (*p* = 0.042) in serum samples of malignant tumors. This miRNA was also shown to be up-regulated in colon, hepatocellular, gastric cancer and malignant melanoma.^24^, ^25^ In the study of Liu et al.,^26^ miR-31 was up-regulated in oral SCC and levels were decreased after treatment, regarding possible role of this miRNA as a marker in this cancer. There was a 2244 fold (*p* = 0.0208) up-regulation of this miRNA in malignant salivary tumor group in tissue samples in our study. miR-146b was shown to be important prognostic factor for papillary thyroid cancer patients, more expression of this miRNA in tumor cells has decreased survival.^27^ miR-146b expression was also increased in anaplastic thyroid cancer cells.^28^ However for breast cancer, expression of miR-146b has caused reduction in metastatic ability by suppressing NF-kB activity.^29^ In our study there was a 3.21 fold up-regulation (*p* = 0.013) of miR-146b in malignant tumor group compared to benign group in tumor tissue specimens. miR-345 was found to have a prognostic value in prostate^30^ and colorectal^31^ cancer. In our study, it was 18 fold up-regulated (*p* = 0.041) in malignant group. For miR-30e; it was shown to be up-regulated in hepatocellular cancer^32^ whereas down-regulated in anaplastic thyroid cancer.^33^ In our study miR-30e showed 15.06 fold up-regulation (*p* = 0.034) in malignant tumor group's plasma samples compared to benign group, whereas there was a down-regulation in benign tumor group compared to control group (*p* = 0.0007) in plasma samples.

Studies concerning the relationship between salivary gland tumors and miRNAs are limited. Mitani et al. studied miRNA profiles of ACC specimens compared to normal salivary tissues. They found that over-expression of miR-17 and miR-20a was related to poor outcome.^34^ He et al. found that expression of miR-181a in ACC tumor cells decreased metastatic potential of the tumor.^4^ Liu et al. speculated that suppression of miR-155 can inhibit cell proliferation and tumor growth for ACC.^35^ Chen et al. found that 17 different miRNAs had statistically significant fold changes in the metastatic ACC compared to non-metastatic ones in cell lines.^5^ Among benign salivary gland tumors pleomorphic adenomas were investigated for possible role of miRNAs in pathogenesis. Zhang et al. found that 17 different miRNAs were up-regulated in tumor samples including miR-21.^36^ This result was not consistent with our study. In our study; there was a down regulation of miR-21 in serum samples of benign tumor group patients compared to control group. This difference may arise from two points. Firstly; our benign tumor group composed of multiple pathologies, not only pleomorphic adenoma. Secondly; serum samples were studied in the control group instead of tissue samples. In another study, Veit et al. compared the miRNA profiles of ACC and SCC of the head and neck region. miR-214, 125a, 574, 199a/b-3p and 199a-5p were up-regulated in ACC group.^37^ miR-199a was also up-regulated in malignant group tissue samples in our study. Recently Kiss et al.^38^ demonstrated possible role of down-regulation of miR-let-7b and miR-193b in salivary gland ACC pathogenesis. Again Kiss et al.^39^ found seven overexpressed miRNAs and nine down-expressed miRNAs in salivary ACC tissue samples in another study. Previously published studies with comparison of our study were summarized in Table 4.

**Table 4:** Previously published studies concerning the relationship between MicroRNAs and salivary gland tumors.

Author	Published year	Tumor type	Study design	Result
Zhang et al.^36^	2009	Pleomorphic adenoma	Pleomorphic adenoma tissue samples were compared with normal salivary gland tissue	Several microRNAs had dysregulations in tumor group.
Liu et al.^35^	2012	ACC	ACC tissue samples were compared with pleomorphic adenoma and normal tissues by using cell culture.	miR-155 is up regulated in ACC cells and causes proliferation, tumor growth and increases invasion
Mitani et al.^34^	2013	ACC	Tumor tissue samples were compared with normal salivary gland tissue	miR-17-92 cluster expression is related to poor outcome
He et al.^4^	2013	ACC	Cell culture of metastatic cell lines were inoculated to mice	miR-181a suppresses metastasis in ACC
Chen et al.^5^	2014	ACC	Cell cultures were obtained from metastatic and non-metastatic cell lines of ACC	17 microRNAs had dysregulation in metastatic cell lines.
Veit et al.^37^	2015	ACC	ACC tissue samples were compared with SCC tissues.	miR-214, 125a, 574, 199a/b-3p, 199a-5p were up-regulated in ACC.
Kiss et al.^39^	2015	ACC	ACC tissue samples were compared with normal salivary gland tissue	7 microRNAs were upregulated and 9 were downregulated.
Kiss et al.^38^	2015	ACC	ACC tissue samples were compared with normal salivary gland tissue	miR-let-7b and miR-193b were down regulated in tissue samples
Cinpolat et al.^a^		Benign and malignant SGTs	(1) Comparison of saliva and serum samples between benign tumors, malign tumors and control group. (2) Comparison of tissue samples between benign and malignant SGT group.	Dysregulation in expression of different types of microRNAs occur according to biological behavior of the SGT. Mir-21and 30e are down-regulated in benign SGT group, up-regulated in malignant ones.

ACC, adenoid cystic carcinoma; SGT, salivary gland tumors; miR, microRNA.

a Presented study.

In our study, we found that miR-21 and miR-30e were up-regulated in the malignant SGT group and down regulated in the benign group. When these miRNAs are investigated for their target genes; RPS7 and LIMCH1 are the common target genes of these miRNAs. (These genes were searched using Mirwalk and Mirtarbase microRNA database systems.)^40^, ^41^ RPS7 gene found on chromosome 2, codes a ribosomal protein belonging to S7E family, and LIMCH1 gene, found on chromosome 4, codes zinc ion-binding proteins.^40^, ^41^ With future studies, zinc ion-binding proteins and ribosomal proteins belonging to S7E family may be new target molecules for differing biological behavior of the salivary gland tumors in serum samples and/or fine needle aspiration biopsy results.

Although our malignant tumor sample size seems to be small, there have been published articles investigating the roles of miRNAs in SGT carcinogenesis with similar sample size. In the study of Veit et al.^37^ they had comparison of 5 tumor tissue samples of ACC with 10 tumor tissue samples of SCC. In the study of Liu et al.^35^ they compared 10 cases of ACC with 4 cases of pleomorphic adenoma and 8 normal parotid gland tissues. Recently Kiss et al.^39^ published their study with only two cases of salivary ACCs. We think that our results are meaningful for investigation of miRNA pathogenesis in SGTs.

## Conclusion

As a result; under the light of previously published articles and current study; we could speculate that miRNAs may have a role in salivary gland tumor pathogenesis. Dysregulation of miRNA type differs according to the biological behavior. miR-21 and miR-30e may have a critical role in salivary gland tumor development with targeting RPS7 and LIMCH1 genes.

## Conflicts of interest

The authors declare no conflicts of interest.
